# Is it Worth Starting Sexual Rehabilitation Before Radical Prostatectomy? Results From a Systematic Review of the Literature

**DOI:** 10.3389/fsurg.2021.648345

**Published:** 2021-04-21

**Authors:** Nadja Schoentgen, Gianluigi Califano, Celeste Manfredi, Javier Romero-Otero, Felix K. H. Chun, Idir Ouzaid, Jean-François Hermieu, Evanguelos Xylinas, Paolo Verze

**Affiliations:** ^1^Department of Urology, Bichat Claude Bernard Hospital, Paris, France; ^2^Department of Neurosciences, Reproductive Sciences, Odontostomatology, University of Naples Federico II, Naples, Italy; ^3^Department of Urology, Instituto de Investigation Sanitaria Hospital 12 de October (imas12), Hospital Universitario 12 October, Madrid, Spain; ^4^Department of Urology, University Hospital Frankfurt, Goethe University Frankfurt, Frankfurt, Germany; ^5^University of Paris, Paris, France; ^6^Department of Medicine, Surgery, Dentistry “Scuola Medica Salernitana”, University of Salerno, Salerno, Italy

**Keywords:** prehabilitation, sexual rehabilitation, sexual dysfunction, radical prostatecomy, prostate cancer

## Abstract

**Background and Purpose:** Sexual dysfunction (SD) is a frequent side effect associated with radical prostatectomy (RP) for prostate cancer (PCa). Some studies have showed the benefit associated with preoperative sexual rehabilitation (prehabilitation) and Enhanced Recovery After Surgery (ERAS) for RP, but no clear clinical recommendations are available yet. Our aim was to conduct a systematic review on sexual prehabilitation prior to RP for patients with a localized PCa and analyze the impact on postoperative sexual health compared with the standard post-operative care.

**Methods:** We performed a systematic review of the literature following the Preferred Reporting Items for Systematic Review and Meta-Analysis (PRISMA) recommendations.

**Results:** Four randomized control trials and one retrospective comparative study were included in the analyses. Three of the five studies showed an improved EF recovery post-RP in the prehabilitation group compared to the standard of care represented by: higher International Index of Erectile Function 5 score (IIEF5) or IIEF score (*p* < 0.0001) and a higher percentage of patients reporting return of EF based on the Sexual Encounter Profile (SEP) (56 vs. 24%, *p* = 0.007). Self-confidence, therapeutic alliance, and adherence to treatment were stronger for patients with preoperative consultations (*p* < 0.05) and EF recovery was better in cases of a higher number of follow-up visits (OR 4–5 visits vs. 1:12.19, *p* = 0.002).

**Discussion:** Despite heterogenous methods and high risks of bias in this systematic review, starting sexual rehabilitation prior to surgery seems to ensure better EF recovery. This prehabilitation should include information of both the patient and his or her partner, with a closer follow up and the use of a multimodal treatment approach that still remains to be defined and validated (oral medication, vacuum devices, pelvic floor muscle training, etc.).

## Introduction

Sexual dysfunction (SD) is a frequent side effect associated with radical prostatectomy (RP) for prostate cancer (PCa). In the Prostate Testing for Cancer and Treatment (ProtecT) trial, which randomized 1,643 patients in three treatments groups (active surveillance, radiotherapy, surgery) and followed-up for 6 years, surgery was associated with the worst rate of SD. At baseline, 67% of men reported erections firm enough for intercourse and this rate declined to 17% at 6 years (1). The lack of preoperative information on postoperative SD can lead to patient and couple distress (2). An approach in which the patient receives adequate treatment and information even prior to surgery seems to improve the rehabilitation phase following the surgical (3, 4) phase, which draws a lot of focus in order to improve the functional outcomes of the surgery. Enhanced recovery after surgery (ERAS) protocols have demonstrated their efficacy for bladder cancer surgery in randomized controlled trials and prehabilitation programs have also been proven to be effective in terms of a faster functional recovery (5, 6). Some studies showed the benefit of prehabilitation and ERAS for RP; however, they mostly focused on blood loss, length of stay, costs, and urinary continence (5, 7). Many studies are published on SD and its treatment after RP (8, 9), but there are really spare data on sexual prehabilitation and its potential impact on postoperative sexual function.

Our aim was to conduct a systematic review on sexual prehabilitation prior to RP for patients with localized PCa and analyze the impact on postoperative sexual recovery compared with the standard post-operative care.

## Methods

### Review Question

According to the Participants, Intervention, Comparison, Outcome, and Study design (PICOS) framework (10), the research question was: In patients undergoing RP for PCa (P), what impact does sexual prehabilitation have (I), compared to the standard postoperative care (C), on the sexual function recovery in the first post-operative year (O), as evidenced by the comparative studies (randomized and non-randomized) (S)?

### Inclusion and Exclusion Criteria

The inclusion criteria were as follows: (1) patients with PCa undergoing RP, regardless of a specific surgical approach; (2) studies that analyzed any type of sexual prehabilitation; (3) outcome measure (sexual function assessed by questionnaires, survey, and scale for psychological impact of sexual dysfunction); and (4) comparative studies [Randomized Controlled Trials (RCTs) and Non-Randomized Studies of Interventions (NRSI)].

The exclusion criteria were as follows: (1) patients undergoing RP for indications other than PCa; (2) patients with PCa managed with treatments other than RP; (3) studies not aimed at analyzing the impact of sexual prehabilitation on the postoperative sexual function recovery; and (4) non-comparative studies, literature reviews, editorials, abstracts, or unpublished research.

### Search Strategy

The Preferred Reporting Items for the Systematic Review and Meta-Analysis (PRISMA) recommendations were followed. A systematic review of the literature was performed in November 2020 using the Cochrane Central Register of Controlled Trials (CENTRAL) and MEDLINE (*via* PubMed) databases. The following terms were combined for the search strategy: sexual prehabilitation, prehabilitation, sexual rehabilitation, prostate cancer, and radical prostatectomy. Search results were filtered by language (English), species (human), and publication date (from January 2000 to November 2020). Reference lists of relevant studies were also reviewed. For studies published by the same authors or institutions, only the most relevant study was reported. Two independent authors (N.S. and G.C.) performed title and abstract screening and full-text review, with a third part to arbitrate (P. V.).

### Data Extraction

The following data were extracted from the included studies: study period, study design, number of subjects included, characteristics of intervention and control groups, study protocol, follow-up, sexual outcomes, results, limitations, and risk of bias.

### Outcomes

The primary study outcome was to assess the impact of sexual prehabilitation vs. standard postoperative care on the sexual function recovery using validated questionnaires.

The secondary outcome was the psychological impact analysis of sexual prehabilitation using questionnaires.

### Risk of Bias Assessment

The Risk of Bias in the included studies were assessed using the Jadad and the Methodological Index for Non-Randomized Studies (MINORS) scores for randomized and non-randomized studies, respectively (11, 12).

### Data Synthesis

Due to the low number of studies included and the high data heterogeneity, we chose not to perform a meta-analysis.

## Results

**Figure 1:**
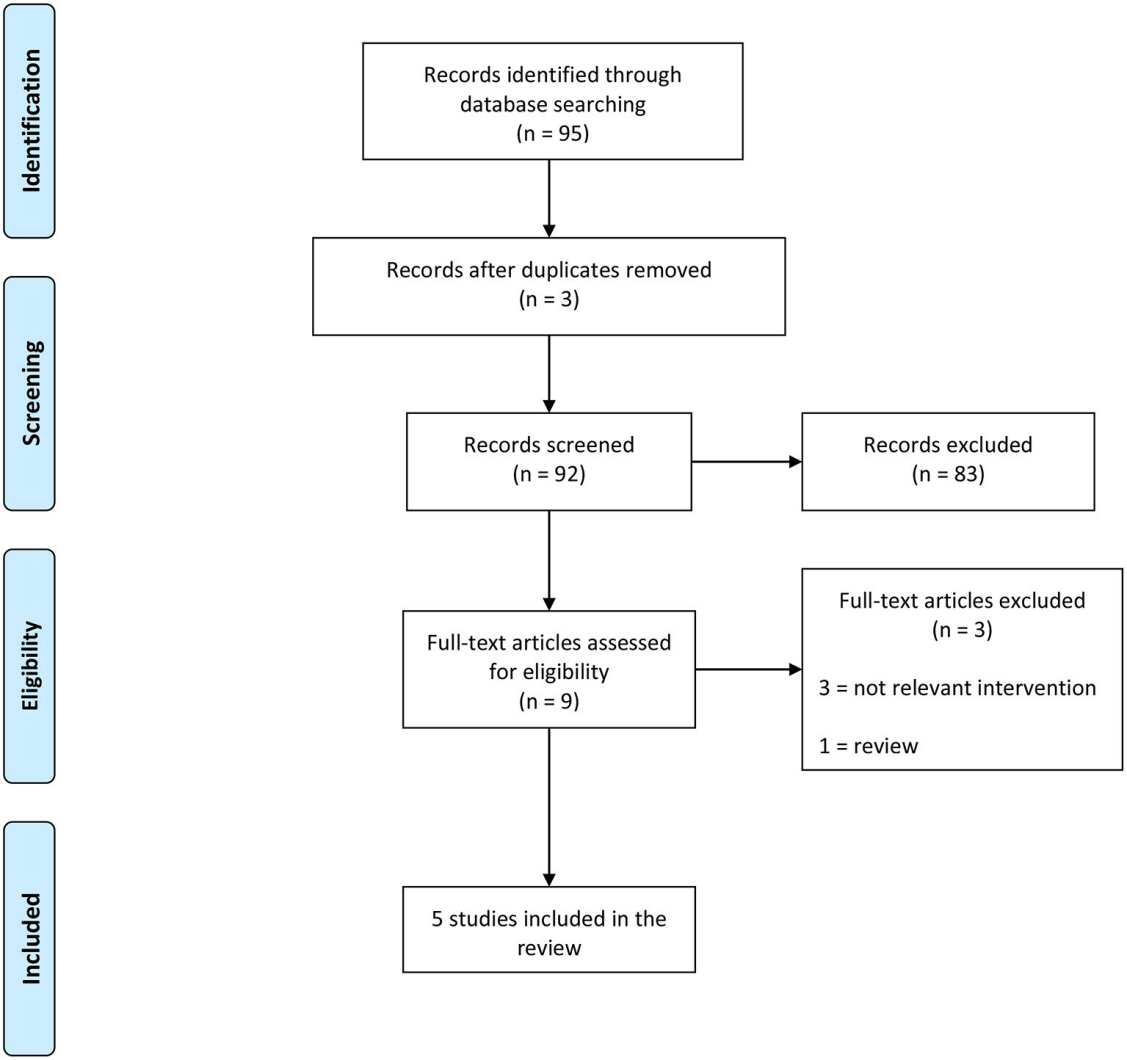
PRISMA flowchart.

We screened 92 studies and included five of them that met the inclusion criteria: four RCTs (13–16) and one retrospective comparative study (17), published between 2015 and 2020. The diagram of the studies' selection is displayed in Figure 1.

The study designs are summarized in Table 1. The number of subjects varied between 31 and 189, and one study included the patient's partner (13). One study protocol was based on counseling (with an additional DVD information tool) (13). In the intervention group, counseling content was education about prostate cancer, menopause, and sexuality; behavioral homework including increasing expression of affection and non-demanding sexual touch; challenging negative beliefs about prostate cancer, aging, and sexuality; and helping the couple choose a medical treatment for erectile dysfunction (ED) and integrating this into their sexual relationship. Three study protocols were based on pelvic floor muscle training (PFMT): in one case, the study only involved the use of total body exercise before RP (15); in the other two studies, the study plan included a pre- and post-intervention treatment (14, 16).

**Table 1:** Summary of study design.

**References**	**Study period**	**Design**	***N***	**Intervention group**	**Intervention group**	**Protocol for intervention group**	**Control group**	**Follow up**	**Sexual outcomes**	
Chambers et al. (13)	May 2009 –May 2011	RCT	*N* = 189 patients and wife (74% recruited pre-surgery)	Peer support volunteers-delivered intervention (*n* = 63)	Nurse-delivered intervention (*n* = 62)	−2 calls prior RP and 6 after for pre-surgery recruited patients - 5 calls post-RP for post-surgery recruited patients - + DVD support	Usual post-RP care	12 months	IIEF	- A scale assessing couples obtention of ED medical help - Psychological Impact of Erectile Dysfunction scale - Masculine Self-Esteem scale - Revised Dyadic Adjustment Scale to assess marital satisfaction - Supportive Care Needs Survey for couples
Santa Mina et al. (15)	February 2014–September 2015	RCT	*N* = 86	Preoperative total body exercise + PFMT (*n* = 44)	-	60 minutes of exercise 3-4 days per week + daily PFMT	Pre-RP PFMT	6 months	IIEF5	-
Lira (14)	March 2013 -December 2014	RCT	*N* = 31	Pre- and post-operative PFMT (*n* = 16)	-	2 preoperative sessions guided by a physical therapist + pre-RP and post-RP PFMT 3/day	Usual post-RP care	3 months	IIEF5	-
Osadchiy et al. (17)	January 2016 - December 2017.	Retrospective comparative study	*N* = 131	Oral therapy + lifestyle counseling before RP + Vacuum post-RP (*n* = 106)	-	5mg tadalafil daily and 1500 mg L-citrulline twice daily + lifestyle counseling 2 weeks before RP and vacuum daily initiated 1-month post-RP	Oral therapy and vacuum initiated 1-month post-RP	12 months	SEP: Q2 and Q3	-
Milios et al. (16)	2016-2018	RCT	*N* = 97	Intensive PFMT pre- and post-RP	-	5 weeks prior and 12 weeks post-RP, intensive PFMT (120 contractions/day instead of 30)	“Standard” PFMT pre- and post-RP	3 months	IIEF5	EF domain of EPIC-CP

*RCT, randomized control trial; N, number of subjects; RP, radical prostatectomy, IIEF, international index of erectile function; ED, erectile dysfunction; PFMT, pelvic floor muscle training; IIEF 5, simplified IIEF 5 items; SEP, sexual encounter profile; EF domain of EPIC-CP, erectile function domain of the expanded prostate cancer index composite for clinical practice*.

One study protocol was based on a combination of oral therapy, lifestyle counseling, and the continuous use of a vacuum device. Intervention group received 5 mg of tadalafil daily and 1,500 mg of L-citrulline twice daily, + lifestyle counseling 2 weeks before RP, and vacuum daily initiated 1 month post-RP (17).

Surgical technique of RP was not mentioned in the studies by Chambers and Lira (13, 14). Osadchiy et al. included only Nerve Sparing Robotic Assisted Laparoscopic Prostatectomy (NS-RALP) without details on uni or bilateral NS surgery (17). Santa Mina et al. included RALP (81%) and open RP (19%) without details on nerve preservation (15). Milios et al. included RALP [87%] and open RP [13%] with unilateral NS surgery (18%), bilateral NS surgery (77%), and non-NS surgery (5%) (16).

Follow-up ranged between 3 and 12 months.

Sexual outcomes were assessed by the International Index of Erectile F unction (IIEF) in one study (13), the short form 5-item IIEF (IIEF-5) in three studies (14–16), question 2 and 3 of the sexual encounter profile (SEP) questionnaire in one study (17), and EF domain of the Expanded Prostate Cancer Index Composite for Clinical Practice (EPIC-CP) in one study (16). In the study by Chambers et al. based on the counseling protocol, psychological scale and couple's assessment were also used.

Functional results derived from all the included studies are summarized in Table 2.

**Table 2:** Summary of study results.

**References**	**Results**			**Limitations**	**Risk of bias (MINORS)**	**Risk of bias (Jadad score)**
Chambers et al. (13)	participants in the peer and the nurse groups were 3.14 times and 3.67 times more likely to use medical treatment for ED respectively than those in the usual care group (*p* = 0.016 and *p* = 0.008)	Men and their partner reported greater therapeutic alliance in the nurse group	Significant higher IIEF (*p* < 0.0001) and greater sexual self-confidence (*p* < 0.05) were associated with patients recruited before RP	Heterosexual couples only included		1
Santa Mina et al. (15)	EF scores were greater in control group at 4-weeks post-RP (3.83 ± 1.33, *p* = 0.004) but not at any other time point			No control group with usual care and short follow up		2
Lira (14)	Tendency toward lower scores of IIEF5 in the Control Group (58.3%) than in the Physical Therapy Group (52.7%) (p = 0.745)			Short follow up, small population		2
Osadchiy et al. (17)	At 12 months, a higher percentage of men in the prehabilitation group reported return of EF compared with the post-RP rehabilitation group (56% [59/106] vs. 24% [6/25], *p* = 0.007)	Patients were more likely to report return of EF if : - they were in the prehabilitation group (OR 4.89, *P* = 0.012) - they underwent bilateral NS-RARP (OR 3.53, *P* = 0.032) - they had more follow-up visits (OR 4–5 visits: 12.19, *p* = 0.002)		Retrospective nonrandomized study, only nerve sparing surgery	10	
Milios et al. (16)	Rates of improvement, supported by reductions in EPIC-CP EF scores and increases in IIEF-5 scores, at 2, 6 and 12 weeks, occurred for patients in both groups with no significant differences between the two groups			No control group with usual care and short follow up		1

*ED, erectile dysfunction; p, probability value; IIEF, international index of erectile function; MINORS, non-random study methodology index; EF, erectile function; RP, radical prostatectomy; p, probability value; IIEF5, simplified IIEF 5 items; NS-RALP, nerve sparing robotic assisted radical prostatectomy; OR, odd ratio; EPIC-CP EF score, erectile function score of the expanded prostate cancer index composite for clinical practice*.

The RCT study by Chambers et al. showed that participants in the peer and nurse groups were 3.14 times and 3.67 times more likely to use medical treatment for ED, respectively, than those in the usual care group (*p* = 0.016 and *p* = 0.008). In this study, a significantly higher IIEF (*p* < 0.0001) and greater sexual self-confidence (*p* < 0.05) were associated with patients recruited before RP (13). The RCT study by Santa Mina et al. concluded that EF scores were greater in the control group at 4-weeks post-RP (3.83 + 1.33, *p* = 0.004) but not at any other time point (15). The RCT study by Lira et al. showed a tendency toward lower scores of IIEF5 in the control group (58.3%) than in the physical therapy group (52.7%) (*p* = 0.745) (14). The RCT study by Milios et al. concluded that rates of improvement, supported by reductions in EPIC-CP EF scores and increases in IIEF-5 scores at 2, 6, and 12 weeks, occurred for patients in both groups with no significant differences between the two groups (16). The retrospective comparative study of Osadchiy et al. showed that at 12 months, a higher percentage of men in the prehabilitation group reported the return of EF compared with the post-RP rehabilitation group [56% (59/106) vs. 24% (6/25), *p* = 0.007] (17). This study also showed that patients were more likely to report the return of EF if: they were in the prehabilitation group (OR 4.89, *P* = 0.012) and if they had more follow-up visits (OR 4–5 visits vs. one visit: 12.19, *p* = 0.002).

The four RCTs presented a Jadad score <3 and the retrospective comparative study a MINOR score of 10.

Regarding the study based on counseling, participants in the peer and the nurse groups were 3.14 times and 3.67 times more likely to use medical treatment for ED, respectively, than those in the usual care group (*p* = 0.016 and *p* = 0.008). In this study, 74% were recruited before RP and a significantly higher IIEF (*p* < 0.0001) and greater sexual self-confidence (*p* < 0.05) were associated with those patients recruited before surgery.

Regarding the three studies using PFMT, none showed significant results but two showed tendencies to a better IIEF5 in the intervention group.

Regarding the study based on oral medication, vacuum, and counseling, a higher percentage of men in the prehabilitation group reported the return of EF compared with the control group [56% (59/106) vs. 24% (6/25), *p* = 0.007].

## Discussion

Guidelines for perioperative care after radical cystectomy for bladder cancer are already published, and strongly recommend that patients should receive routine dedicated preoperative counseling and education (18). Prehabilitation programs have also been proven to be effective in terms of a faster functional recovery (5). No guidelines are available for RP yet but there is a need for patients to be better prepared prior to surgery in order to minimize side effects especially at the time of minimally invasive surgery and ERAS. To the best of our knowledge, this review is the first one focussed on sexual prehabilitation before RP. We highlighted two important aspects: (1) three of the five papers showed better EF recovery post-RP if patients received a pre-surgical care; (2) self-confidence, therapeutic alliance, and adherence to treatment were stronger for patients with preoperative consultations and EF recovery was better in cases of a higher number of follow-up visits.

Age and preoperative EF are the most important predictors for better postoperative sexual outcomes (19). Preservation of the neurovascular bundles during RP may spare EF (20). Nerve-sparing (NS) surgery does not impact oncological outcomes if patients are carefully selected (21, 22). According to the current European Association of Urology (EAU) guidelines, it can be proposed in patients at low risk of extracapsular extension (based on cT stage, ISUP grade, nomogram, and multiparametric MRI) (23). Harris et al. found that the NS technique resulted in better sexual function in most men except in those with a low baseline of sexual function (24). Regarding the surgical technique, (extra-, inter-, and intra-fascial approaches), dissections closer to the prostate and performed bilaterally appear to be associated with better functional outcomes (sexual function and continence) (25–27). Novara et al. demonstrated that age ≤ 60 years, Charlson score of 0, and baseline IIEF-6 score >21 were predictors of EF recovery after NS surgery (28). In view of these results, we can suggest that ensuring a good preoperative sexual potency could improve postoperative sexual recovery and support the fact that prehabilitation should be developed and encouraged.

Despite the introduction and improvement of the NS techniques, ED is still commonly reported after RP (between 14 and 69% of cases) (29). Although a meticulous surgical procedure can be performed to avoid direct injury to the cavernous nerves, ED can occur as a consequence of neuropraxia due to traction, compression, or coagulation (30). In 4–75% of men, an accessory pudendal artery (APA) can run parallel to the dorsal vascular complex. Ligation of APA during RP could have a role in penile hypoxia independent from denervation (30). Promptly after the nerve injury, regardless of the severity and extent, a neuroinflammatory cascade is triggered, which ultimately results in the apoptosis of neurons and degeneration of axons in a process known as the Wallerian degeneration (31). The subsequent denervation of the corpora cavernosa leads to the worsening or loss of daily and nocturnal erections, inducing a persistent state of hypoxia. Penile hypoxia results in fibrosis and smooth muscle cell apoptosis (32). These events lead to a veno-occlusive dysfunction and consequent ED (33). The autonomic nervous system has an inherent capacity to regenerate after nerve injury, mediated by the secretion of neurotrophic factors in response to damage. Nonetheless, this mechanism is generally insufficient to prevent the organ's functional failure (34). Even if nerve sparing (NS) surgery is associated with a better post-operative EF, it is not the only factor to take into account to preserve sexual function.

Only three of the five studies included mentioned the RP surgical technique. A majority of NS-RALP was performed and none of the studies analyzed the impact of surgical technique on sexual outcomes. Unfortunately, we did not have enough data in our review to analyze the implication of RP modalities on the sexual prehabilitation results.

To date, different post-operative sexual rehabilitation strategies are published. Actual treatment options for ED management following RP are: oral therapy with phosphodiesterase type 5 inhibitors (PDE5-I) (35), vacuum devices (36), intra-urethral instillation (37) or intracavernous injections (ICI) of prostaglandin (38), and penile implant (39). The International Consultation for Sexual Medicine (ICSM) 2015 recommendations attest that there are conflicting data as to whether penile rehabilitation with PDE5i improves recovery of spontaneous erections. These recommendations also highlight that the data are inadequate to support any specific regimen as optimal for penile rehabilitation (40). PDE5i inhibit the PDE5 which prolongs action of cyclic guanylate monophosphate (cGMP) which leads to smooth muscle relaxation and erection, but nerve activation is required to initiate cGMP synthesis (41). This explains why only 0 to 15% of men treated by non-NSRP responded to PDE5i vs. 35 to 75% among those treated by NSRP (42). Many studies analyzed the effect of on-demand vs. daily vs. scheduled use of PDE5i, but rehabilitation strategies using PDE5i following RP do not increase self-reported potency and EF compared to on-demand use (9). A recent meta-analysis suggests that the early use of vacuum therapy appears to have a good therapeutic effect on post-RP patients and no serious side effects. Due to the overall limited quality of the included studies, this result needs to be confirmed (43). Intra-urethral alprostadil also appears to be a successful ED treatment after RP (37) and a good alternative in cases of patient refusing oral medication and injection. Despite ICI and penile implant are considered second- and third-line therapies for ED after RP, in the prospective analysis on EF after RP for high risk PCa published by Sridhar et al. 48 patients of the non-NSRP received ICI or penile implant and 94% of men on these treatments returned to baseline IIEF-5 scores. This highlights that men who undergo non-NSRP and consider EF a high priority after surgery should be commenced on immediate second- or third-line therapies because of the low rate of PDE5i efficacy (44). EAU guidelines confirm that data is inadequate to support the use of any specific regimen for penile rehabilitation after RP (35).

New approaches were recently proposed for sexual rehabilitation following pelvic surgery: low intensity extracorporeal shockwave therapy (Li-ESWT) and PFMT. Preliminary studies showed, on rat models, that Li-ESWT resulted in angiogenesis, tissue restoration, and nerve regeneration which facilitated a more complete reinnervation of penile tissue (45). There is no published study on early Li-ESWT after RP for sexual rehabilitation, but positive results were obtained in patients with organic ED (46) and this treatment should be evaluated as an option of sexual pre- and post-RP rehabilitation. Many studies have demonstrated the benefits of PFMT for treating urinary incontinence in men following RP but literature reviews published in 2017 and 2020 also showed its efficacy for post-RP sexual rehabilitation (47, 48). Most studies of these reviews demonstrated improvements in EF with PFMT; however, a lack of methodological rigor and variability among protocols limited the interpretation of results.

Just as there is still no ideal and unambiguous protocol suggested for post-surgical sexual rehabilitation, there is absolutely no evidence regarding the best pre-surgery approach.

Our review showed that PFMT, oral medication, and vacuum started before surgery could be effective on EF recovery but also that information of patient and wife and a closer follow-up seems to be really important in sexual recovery (4). Our study is not devoid of limitations that primarily include the low number of studies, heterogenous protocols with high risk of bias (RCTs with a JADAD score <3 are of poor quality). At the same time, it highlights the need of further research on sexual rehabilitation started before RP. This is why two multimodal sexual prehabilitation protocols have been published and results should be the subject of future publications (49, 50).

The limits of this review are the small number of studies included and heterogeneity of methodology which highlights the lack of literature data on this really important topic and the need to improve our knowledge on sexual RP side effects management.

In conclusion, to try to briefly answer the clinical question of our review today, we do not have a solid scientific evidence to state with certainty what and when it is best to do sexual rehabilitation to obtain the best restoration of sexual function in the patient who undergoes RP. However, we have a few general principles that should be followed in the clinical management of patients and which include: (1) the correct selection of the patient who can really benefit from a NS approach, and that primarily cannot be separated from an optimal erection before surgery; (2) start a therapeutic protocol as soon as possible after the surgery, why not even before the surgery?; (3) use the most effective treatment modality to which the patient adheres best [PFMT seems to be a good treatment option in our review and in reviews already published (47, 48)]; and (4) involve the patient and his or her partner as much as possible in the rehabilitation program, because it is the concrete motivation to do everything possible the prelude to the optimal result. Preoperative and post-operative patient and partner information on sexual side effects and preoperative and postoperative PFMT rehabilitation protocol should be a good way to improve sexual recovery in clinical practice.

## Conclusion

ED remains a frequent side effect after RP and really impact the patients' quality of life. Starting sexual rehabilitation prior to surgery seems to ensure a better post-operative EF recovery. This prehabilitation should include information of the patient and his or her partner, with a closer follow up (possibly with digital information supports), and the use of a multimodal treatment approach (oral medication, PFMT, vacuum devices, Li-ESWT). These protocols need to be tested and validated in a large RCT for stronger evidence.

## Data Availability Statement

The original contributions presented in the study are included in the article/supplementary material, further inquiries can be directed to the corresponding author/s.

## Author Contributions

NS, GC, and PV performed title and abstract screening and full text review. NS wrote the manuscript, and prepared the tables, and figure. GC, CM, JR-O, FC, IO, J-FH, EX, and PV helped with redaction. All authors contributed to the article and approved the submitted version.

## Conflict of Interest

The authors declare that the research was conducted in the absence of any commercial or financial relationships that could be construed as a potential conflict of interest.
